# Transcriptional analysis of micro-dissected articular cartilage in post-traumatic murine osteoarthritis

**DOI:** 10.1016/j.joca.2014.12.014

**Published:** 2015-04

**Authors:** M.D. Gardiner, T.L. Vincent, C. Driscoll, A. Burleigh, G. Bou-Gharios, J. Saklatvala, H. Nagase, A. Chanalaris

**Affiliations:** Kennedy Institute of Rheumatology, Nuffield Department of Orthopaedics, Rheumatology and Musculoskeletal Sciences, University of Oxford, Old Road Campus, Roosevelt Drive, Headington, Oxford, OX3 7FY, UK

**Keywords:** Osteoarthritis, Destabilisation of the medial meniscus (DMM), Microarray, Tenascin, Fibromodulin, Fibronectin

## Abstract

**Objective:**

Identify gene changes in articular cartilage of the medial tibial plateau (MTP) at 2, 4 and 8 weeks after destabilisation of the medial meniscus (DMM) in mice. Compare our data with previously published datasets to ascertain dysregulated pathways and genes in osteoarthritis (OA).

**Design:**

RNA was extracted from the ipsilateral and contralateral MTP cartilage, amplified, labelled and hybridized on Illumina WGv2 microarrays. Results were confirmed by real-time polymerase chain reaction (PCR) for selected genes.

**Results:**

Transcriptional analysis and network reconstruction revealed changes in extracellular matrix and cytoskeletal genes induced by DMM. TGFβ signalling pathway and complement and coagulation cascade genes were regulated at 2 weeks. Fibronectin (*Fn1*) is a hub in a reconstructed network at 2 weeks. Regulated genes decrease over time. By 8 weeks fibromodulin (*Fmod*) and tenascin N (*Tnn*) are the only dysregulated genes present in the DMM operated knees. Comparison with human and rodent published gene sets identified genes overlapping between our array and eight other studies.

**Conclusions:**

Cartilage contributes a minute percentage to the RNA extracted from the whole joint (<0.2%), yet is sensitive to changes in gene expression post-DMM. The post-DMM transcriptional reprogramming wanes over time dissipating by 8 weeks. Common pathways between published gene sets include focal adhesion, regulation of actin cytoskeleton and TGFβ. Common genes include Jagged 1 (*Jag1*), Tetraspanin 2 (*Tspan2*), neuroblastoma, suppression of tumourigenicity 1 (*Nbl1*) and N-myc downstream regulated gene 2 (*Ndrg2*). The concomitant genes and pathways we identify may warrant further investigation as biomarkers or modulators of OA.

## Introduction

Osteoarthritis (OA) is a polygenic, multifactorial disease of synovial joints, characterised by articular cartilage degradation and changes in other joint tissues^1–3^.

The molecular events occurring during disease initiation and early progression are poorly understood. Initial large-scale gene expression studies used osteoarthritic human cartilage from end-stage disease^4–7^. These studies demonstrated changes in gene expression in late stage OA but were unable to examine early molecular events as it is very difficult to procure human cartilage from different stages of disease and pair it with non-diseased age-matched tissue. Animal models provide genetically controlled and aged-matched tissue samples to allow the study of OA pathogenesis^8^. Broad molecular approaches are able to identify single pathogenic molecules and unravel networks of interacting genes/proteins that are dysregulated in disease^9^.

The destabilisation of the medial meniscus (DMM) surgical model of OA is a widely used and validated model of post-traumatic OA^10^. We have used it to examine gene expression profiles shortly after joint destabilisation. We found early gene expression changes are highly mechanosensitive and largely attenuated by joint immobilisation^11^. When analysing the whole joint tissue, immobilised joints do not have increased expression of pathogenic proteases such as *Adamts5*, *Adamts4*, *Adamts1* and *Mmp3*, show attenuated expression of inflammatory response genes like *Ccl2*, *Il1b* and *Il6* and are protected from disease. Similarly, joint immobilisation led to decreased expression of those genes in the cartilage at 6 h post-DMM. These results indicate the rapid response of cartilage to DMM. We now extend these results showing gene changes in this highly mechanosensitive tissue occurring beyond 6 h, over an 8-week course. We were interested in identifying “phases” of disease progression that might provide novel targets and new biomarkers for investigation in the human condition.

## Methods

### Animals and surgical model of OA

All procedures complied with the Animals (Scientific Procedures) Act 1986 and with guidelines of the local ethics committee. C57BL/6J male mice were purchased from Charles Rivers (Margate, UK). Mice were housed 4–5 per standard individually ventilated cages and maintained under 12-h light/12-h dark conditions at an ambient temperature of 21°C. Animals were fed a certified mouse diet (RM3; Special Dietary Systems) and water *ad libitum*. In total 187 animals were used in this study (Supplementary Fig. 1).

**Fig. 1:**
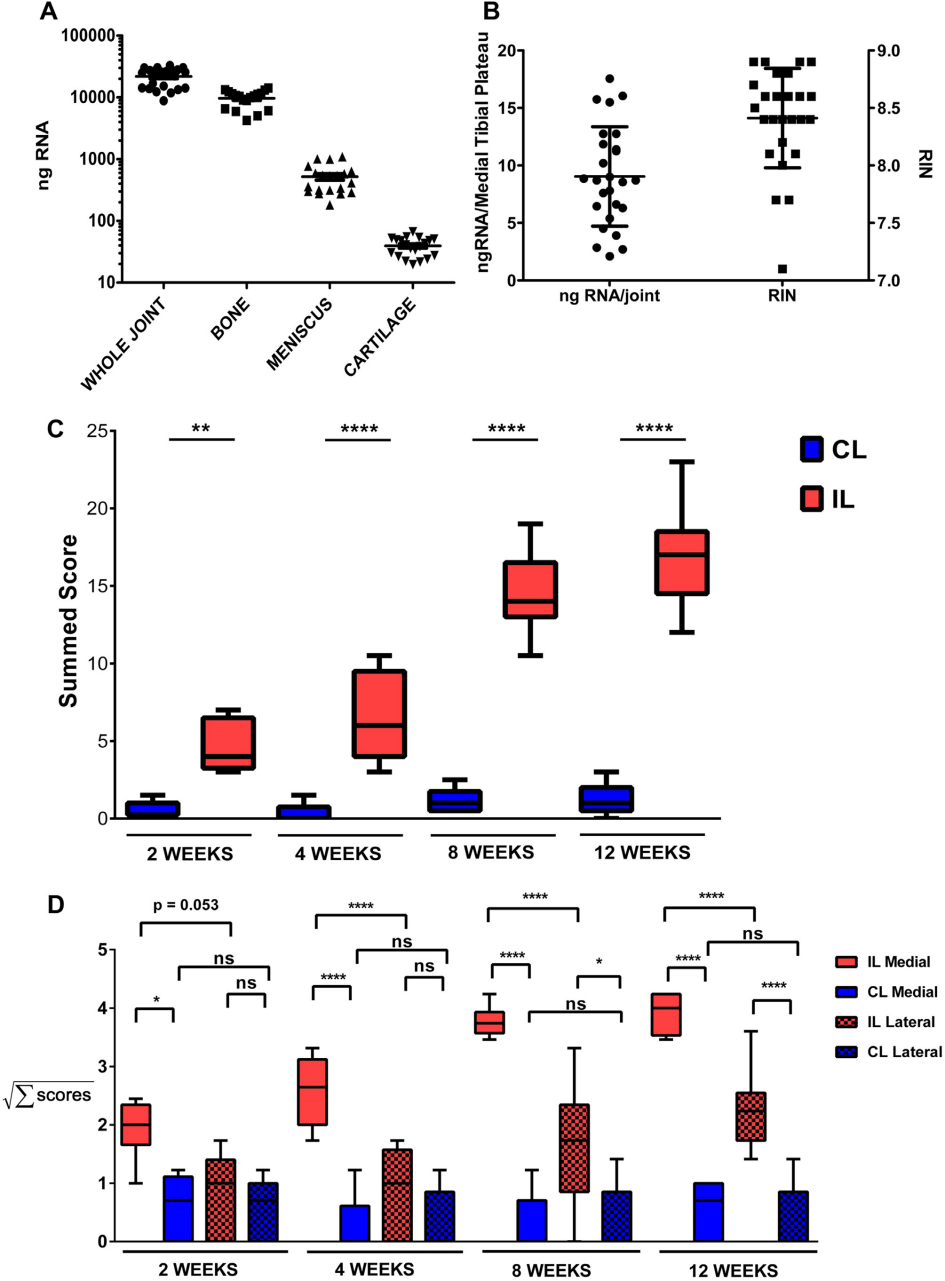
Quantity and quality of RNA isolated from dissected knee tissues and histological scores for cartilage degradation post-DMM. A: Amount of RNA isolated from a whole joint (*n* = 22; 11 animals, 10–14 weeks of age) and from cartilage (*n* = 20 each point is a pool of at least two joints; 30 mice in total, 10–14 years of age; the same group of 30 animals was used for the meniscus and bone RNA isolation), meniscus (*n* = 20) and tibial epiphysis with the cartilage removed (Bone) (*n* = 20). B: Amount per joint and quality of RNA isolated from MTP cartilage (*n* = 29; each point is a pool of 4–8 MTPs, we used 102 mice in total ranging from 10 to 18 weeks old. This group includes the 30 animals used on panel A and the 72 used for the microarray). C: Histological assessment of coronal knee sections at 2, 4, 8 and 12 weeks post-DMM. Boxes represent the summed histopathological score of cartilage degradation of the unoperated (CL, blue boxes) and operated knees (IL, red boxes). For each time point 10 mice were used (30 in total) (*n* = 10). The whiskers cover the breadth of the first to the ninety-ninth percentile of the scores with the median represented by the horizontal line in the box. The values for all groups approximate a Gaussian distribution (D'Agostino and Pearson omnibus normality test, *P* > 0.07). (*: *P* < 0.05, **: *P* < 0.01, ***: *P* < 0.001, ****: *P* < 0.0001 two way analysis of variance (ANOVA), Bonferroni *post-hoc* test, the *P* values are multiplicity adjusted). D: Histological assessment of coronal knee sections at 2, 4, 8 and 12 weeks post-DMM. Boxes represent the summed histopathological score of cartilage degradation of the unoperated (CL, blue boxes) and operated knees (IL, red boxes) on the medial (solid boxes) and lateral (checked boxes) side. For each time point 10 mice were used (30 in total) (*n* = 10). The whiskers cover the breadth of the first to the ninety-ninth percentile of the scores with the median represented by the horizontal line in the box. The data were transformed by taking the square root of the values prior to the statistical testing. After transformation the values for all groups approximate a Gaussian distribution (D'Agostino and Pearson omnibus normality test, *P* > 0.07. (*: *P* < 0.05, **: *P* < 0.01, ***: *P* < 0.001, ****: *P* < 0.0001 two way ANOVA, Bonferroni *post-hoc* test, the *P* values are multiplicity adjusted).

DMM surgery was performed on 10-week-old mice during daylight hours following the protocol described previously in Ref. 11. The right knee (ipsilateral) was operated and the left (contralateral) remained unoperated, acting as a control. For microarray, three groups of eight mice were operated on per time point (Fig. 1). For histological analysis, 10 mice were operated on per time point. For PCR, an extra three groups of five mice were operated on for each time point. An additional 15 mice were used to quantify the amount of RNA extractable from each joint tissue. Eleven of these mice were whole joint extractions and the other four were micro-dissected. No pooling was performed for meniscus or subchondral bone samples. Where pooling was performed, the experimental unit was regarded as one (Supplementary Fig. 1).

### Histological analysis

Knee joints from operated and contralateral limbs were dissected and processed for histological analysis at 2, 4, 8 and 12 weeks. Two blinded independent observers scored the cartilage morphology, as described previously in Ref. 11.

### RNA extraction and whole genome expression analysis (WGA)

Micro-dissection of joint tissues was performed as previously described in Ref. 11 at 2, 4 and 8 weeks following DMM surgery. Detailed protocol is provided in the supplementary file. RNA extraction, amplification, cDNA labelling, hybridisations on an Illumina Mouse WG-6 v2.0 Expression BeadChip (Illumina), microarray processing, data analysis and construction of regulatory pathways was performed as described in the Supplementary File. Data are deposited in NCBI's Gene Expression Omnibus^12^ with GEO Series accession number GSE53857 (http://www.ncbi.nlm.nih.gov/geo/query/acc.cgi?acc=GSE53857).

### Comparison of previously published microarray datasets

We compared our 2-week dataset with other arrays owing to the size of our dataset and availability of other mouse arrays at this time point. We selected previously published datasets fulfilling the following criteria:

a. They were from pure knee cartilage RNA^5–7,13–16^, or enriched for cartilage^17^;
b. They were of mouse^14,17^, rat^13,15^ or human origin^5–7,16^;
c. The rodent sets were from models of surgical induced knee instability; and
d. Where possible we preferred the 2-week time point for comparison^14,17^.

We used the gene-lists corresponding to the significantly dysregulated probes of each study. Additionally, we compared the overlap between our array and a gene-set specific for inflammatory-related genes^18^. Details are found in the Supplementary File. The Venn diagrams were constructed using Venny^19^. Hypergeometric distribution probability mass function (p.m.f.) calculations of overlaps observed between two sets were done in R.

### Real-time (RT)-PCR

RT-PCR was performed using RNA from biological replicates at each time-point as described before in Ref. 11. The list of assays used is available upon request.

## Results

### Determining the relative abundance of articular cartilage RNA in the whole joint

We established a method to isolate RNA from distinct tissues within the mouse knee joint^11^. The tibial epiphysis contributes almost half of the whole joint RNA (44% ± 20%), with lesser contributions from meniscus (2.4% ± 1.5%) and cartilage (0.174% ± 0.082%) [Fig. 1(A)]. Although the articular cartilage contributes a small percentage of total joint RNA, it is clearly implicated in early OA. Gene expression changes localised to this tissue may be lost when taken with the whole joint tissue. The RNA isolation technique resulted in 9 ± 4.3 ng RNA/joint from the pooled medial tibial plateau (MTP) with a RIN of 8.4 ± 0.4 [Fig. 1(B)].

We extended our original observations by performing a microarray analysis of genes regulated specifically in the MTP cartilage beyond 2 weeks.

### Histological cartilage degradation in operated joints relative to controls

Operated joints showed progressive increase in pathological changes over the 12-week time course [Fig. 1(C) and (D)]. Within the operated joints, histological scores were consistently higher in the medial compartment compared to the lateral compartment [Fig. 1(D)]. No significant pathological changes were detected in the contralateral knees and there was no difference between the medial and lateral compartment of the contralateral knees at any time point.

### Gene changes in operated joints relative to controls and network evolution following surgery

Pairwise comparisons of DMM-operated and contralateral MTP cartilage were performed at 2, 4 and 8 weeks (Supplementary Table S1). The number of regulated genes decreased with time following surgery (Supplementary Table S2).

**Fig. 2:**
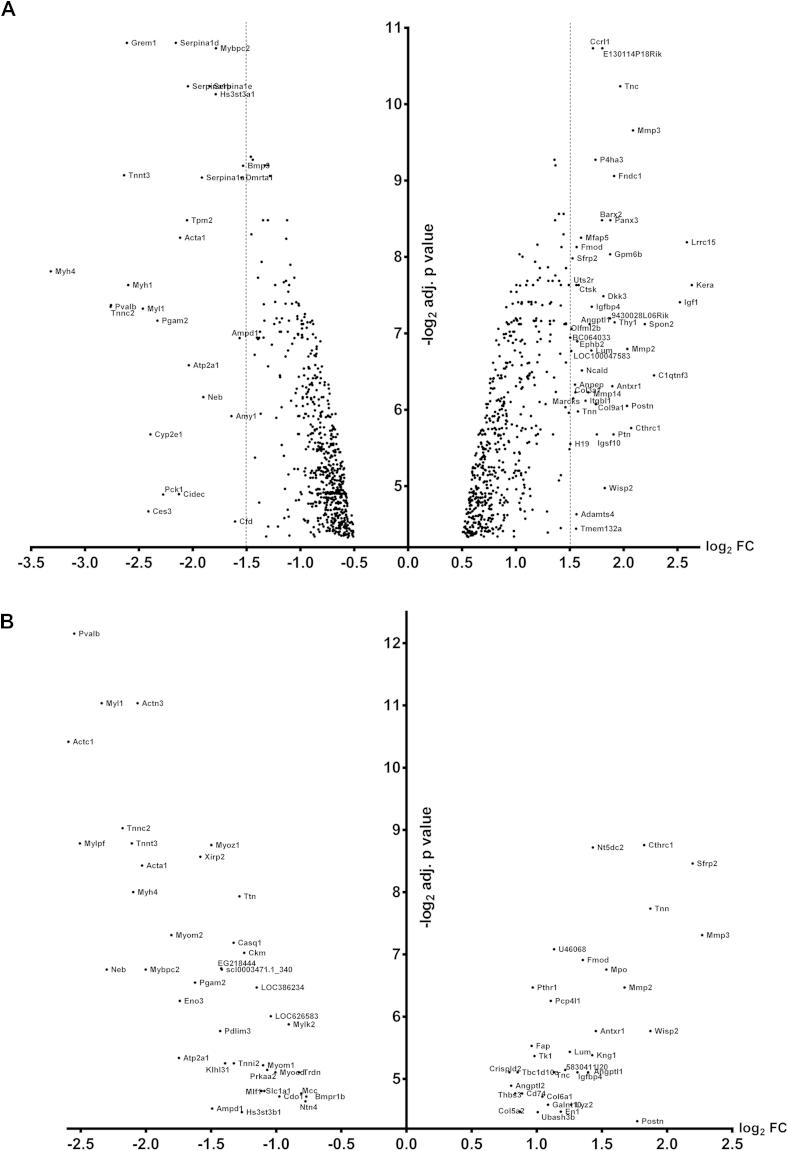
Differentially expressed genes in the operated medial tibial cartilage at 2 (A) and 4 (B) weeks. The base 2 logarithm of the fold change for each gene that was confidently detected in our array was plotted against the base 2 logarithm of the *P* value that was adjusted for multiplicity. The names of the genes with a fold change greater than 2.8 or less than 0.35 are presented at 2 weeks, where the dotted vertical line represents the boundaries for those fold changes. At 4 weeks the significantly regulated genes with a fold change greater than 1.5 and less than 0.5 are represented.

At 2 weeks, 1224 genes (1425 probes) were differentially expressed [Fig. 2(A); Supplementary Table S3]. Keratocan (*Kera*) was the most significantly up-regulated gene, others included *Mmp3*, *Mmp2* along with a further 14 matrix metalloproteinases (MMPs). Fibromodulin (*Fmod*) was one of a number of small leucine-rich repeat proteins (SLRPs) up-regulated. *Myh4* was the most down-regulated gene, along with other contractile elements and many genes from the serpin family [Fig. 2(A)].

At 4 weeks, 76 genes (90 probes) were differentially expressed [Fig. 2(B), Supplementary Table S3] (20 genes were unique for week 4). Genes up-regulated at 2 weeks that were also up at 4 weeks included *Mmp3*, *Thrombospondin 3* (*Thbs3*), *Tenascin N/W* (*Tnn*), *Tenascin C* (*Tnc*) and *Fmod*. Similarly to 2 weeks, many genes for contractile elements were down-regulated. At 8 weeks, only *Tnn* and *Fmod* were up-regulated (Supplementary Table S3); no genes were down-regulated. Pairwise comparisons of control joints at 2, 4 and 8 weeks showed no difference in gene expression.

**Fig. 3:**
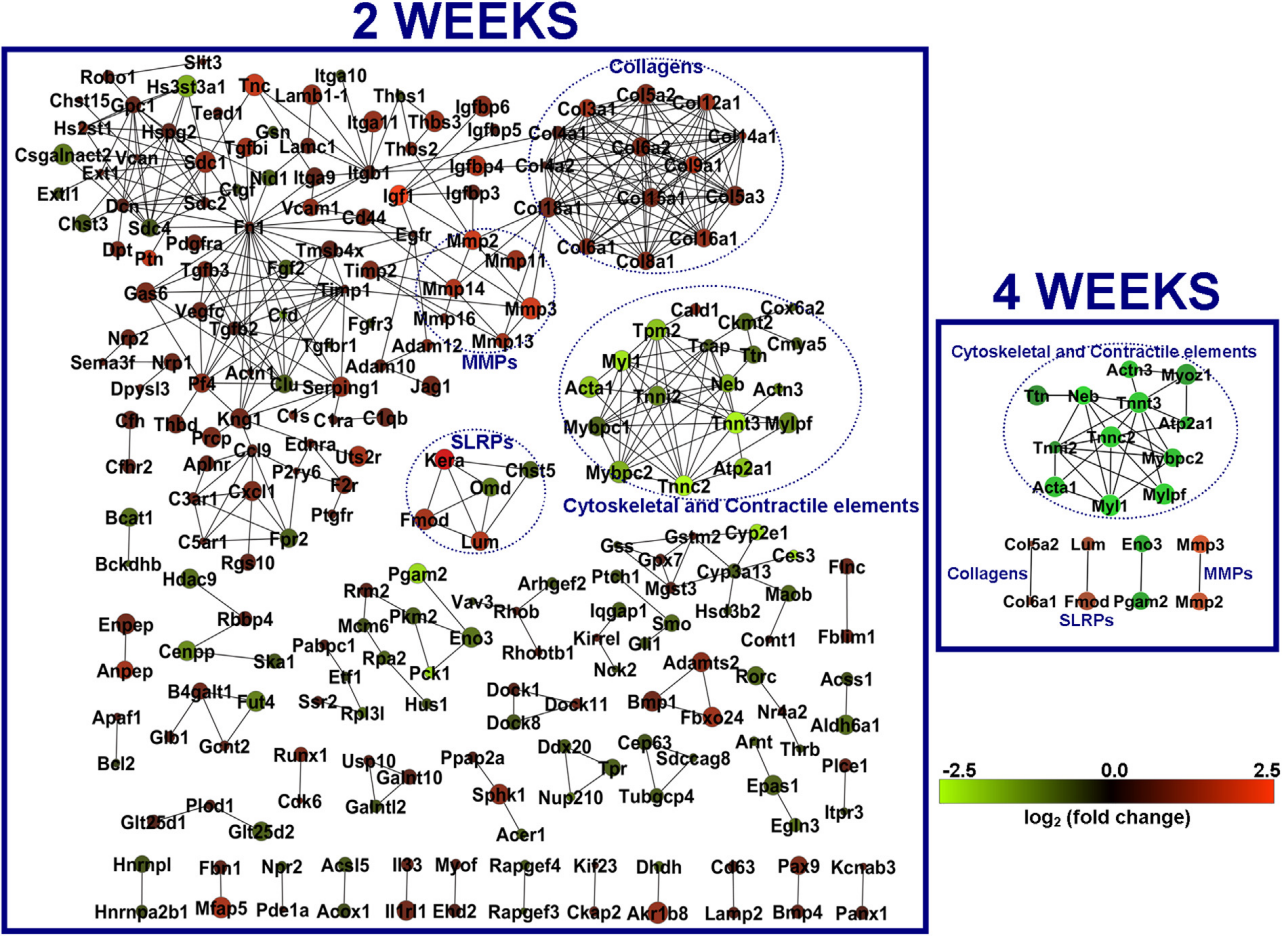
Network representation of the protein–protein interactions between genes that were significantly regulated at 2 (right panel) and 4 weeks (left panel). Only genes with at least one interacting partner are represented. Nodes are coloured according to the fold change in gene expression over the contralateral limbs. Green represents down-regulated components and red represents up-regulated genes. The gradient bar shows the change in colour according to the fold change. The size of the node represents the significance of the observed change (adjusted *P* value for pairwise comparisons) with smaller nodes closer to 0.05 and increasing size towards an adjusted *P* value of 0. The dotted blue circles highlight group of nodes in the 2 week network that has at least one connected member in the 4 week network. The titles of the circles, represent the functional group of the circled nodes (MMPs: matrix metalloproteinases, SLRPs: small leucine repeat proteins).

Network analysis was performed using STRING and EGAN. Each node represents a protein and the edges their regulatory relationships. Multi-edged nodes are described as hubs. At 2 weeks, 424 edges were observed between 233 nodes (Fig. 3). The interactions revealed a number of hubs at the 2-week time point. Fibronectin (*Fn1*) was the node with the highest number of edges, thus placing it at the centre of the biggest network. Next was a cluster of collagens with at least 10 edges each. Other nodes with at least 10 edges were the troponins, clusterin, decorin, syndecans 1 and 4 (*Sdc 1* and *4*), integrin β1 (*Itgb1*), perlecan (*Hspg2*), transforming growth factor β2 (*Tgfb2*) and glypican 1 (*Gpc1*). At 4 weeks, 32 edges were observed between 20 nodes (Fig. 3). The main network was clustered around troponin T3 (*Tnnt3*); four sub-networks had a single edge each.

### Functional annotation clustering (FAC) analysis

To gain further insight into the biological significance of the gene expression changes KEGG pathway analysis (Supplementary Table S4) and FAC was performed (Supplementary Tables S5–S7). At 2-weeks, enriched KEGG pathways included ECM-receptor interaction (*P* < 0.0001) and focal adhesion (*P* < 0.0001), which contained collagens, tenascins, integrins and thrombospondins. The pathways for hypertrophic cardiomyopathy (*P* = 0.005), TGF-β signalling (*P* = 0.006), and dilated cardiomyopathy (*P* = 0.008) contained genes for integrins, IGF-1 and TGF-β2 and -β3. Complement and coagulation cascades (*P* < 0.0001) and regulation of actin cytoskeleton (*P* = 0.002) were particularly enriched in down-regulated genes. O-glycan and heparan sulphate biosynthesis pathways (*P* = 0.01 and *P* = 0.04, respectively), and galactose metabolism (*P* = 0.04) are present. FAC analysis (Supplementary Table S5) confirmed the significant presence of metalloproteinases (clusters 1, 6 and 9), collagens and thrombospondins (cluster 2). Down-regulated genes (Supplementary Table S6) showed involvement in cytoskeletal organisation (clusters 1, 2, and 4) and apoptosis (clusters 3 and 9).

At 4 weeks, KEGG analysis showed enrichment for ECM-receptor interaction (*P* < 0.0001) and focal adhesion (*P* < 0.0001) (Supplementary Table S4). In common with the 2-week time point cytoskeletal elements were down-regulated. The KEGG pathway for hypertrophic cardiomyopathy was enriched (*P* = 0.022); it contained the genes *Actc1*, *Prkaa2* and *Ttn*. These genes did not feature at 2 weeks. FAC confirmed the dominance of cytoskeletal elements (Supplementary Table S7).

### RT-PCR verification of gene expression changes

**Fig. 4:**
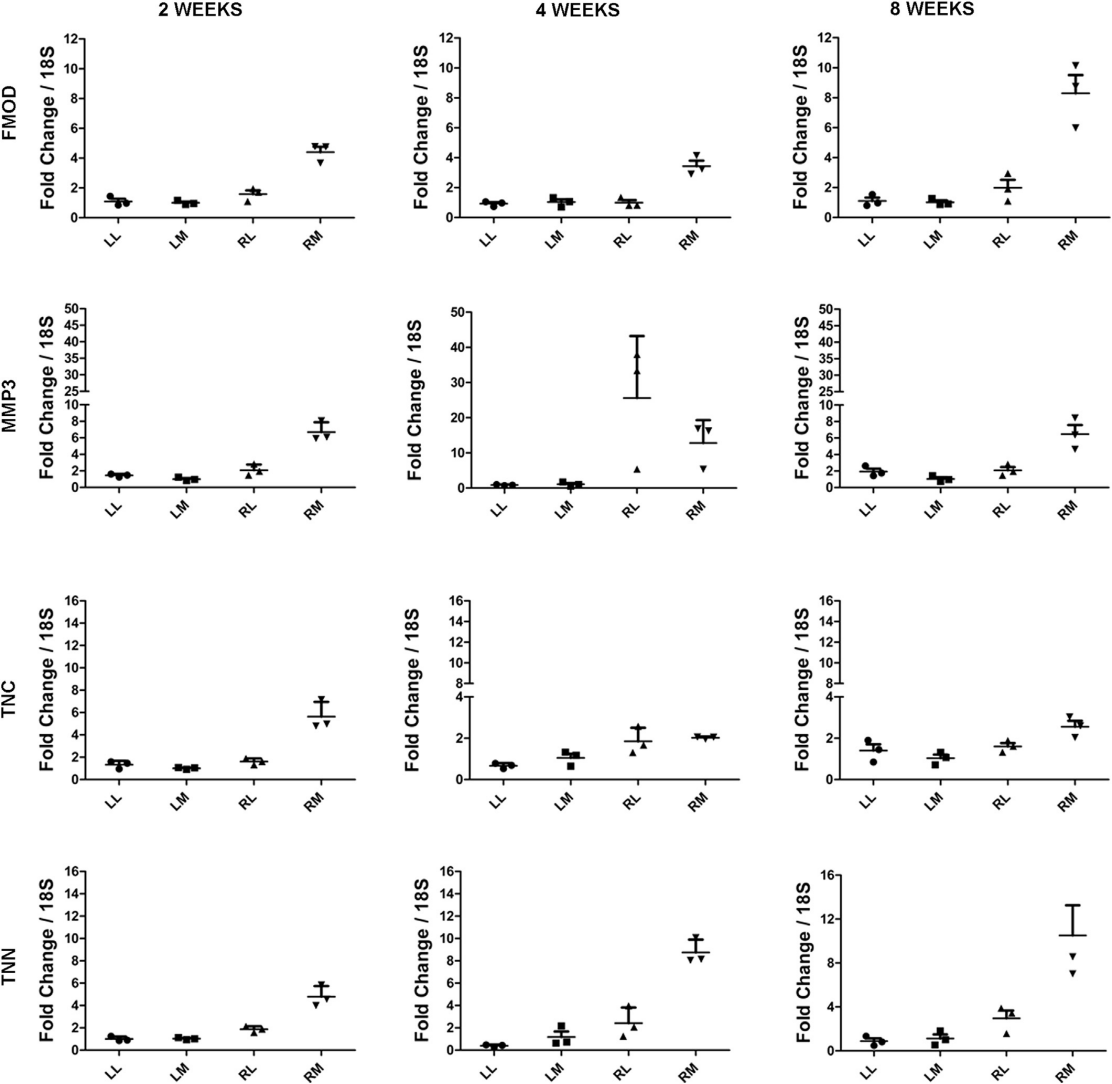
Quantitative RT-PCR demonstrates that the selected genes are regulated mainly on the MTP cartilage. LL: contralateral lateral, LM: contralateral medial, RL: ipsilateral lateral, RM: ipsilateral medial. [*n* = 3 and each point is a pool of five cartilage compartments; therefore 45 mice were used; data from 30 of these mice were used for the data on Fig. 1(A) and (B) as well.]

We selected four genes to evaluate based on those that were significantly changed at all time points (*Fmod* and *Tnn*) or are of known importance in OA (*Mmp3*); finally *Tnc* was selected as it is a homologue of *Tnn* and was not significantly regulated at 8 weeks. We evaluated cartilage from the medial and lateral plateaus of the operated and contralateral joints at 2, 4 and 8 weeks following surgery (Fig. 4). Gene expression in the lateral plateaus from the operated and contralateral joints was also evaluated by RT-PCR (Fig. 4). *Fmod*, *Tnn* and *Mmp3* (except 4 weeks) were up-regulated in the medial plateau of the DMM joints compared with the lateral plateau and contralateral joint, suggesting very localised response to injury.

### Comparison of our dataset with previously published microarrays

**Fig. 5:**
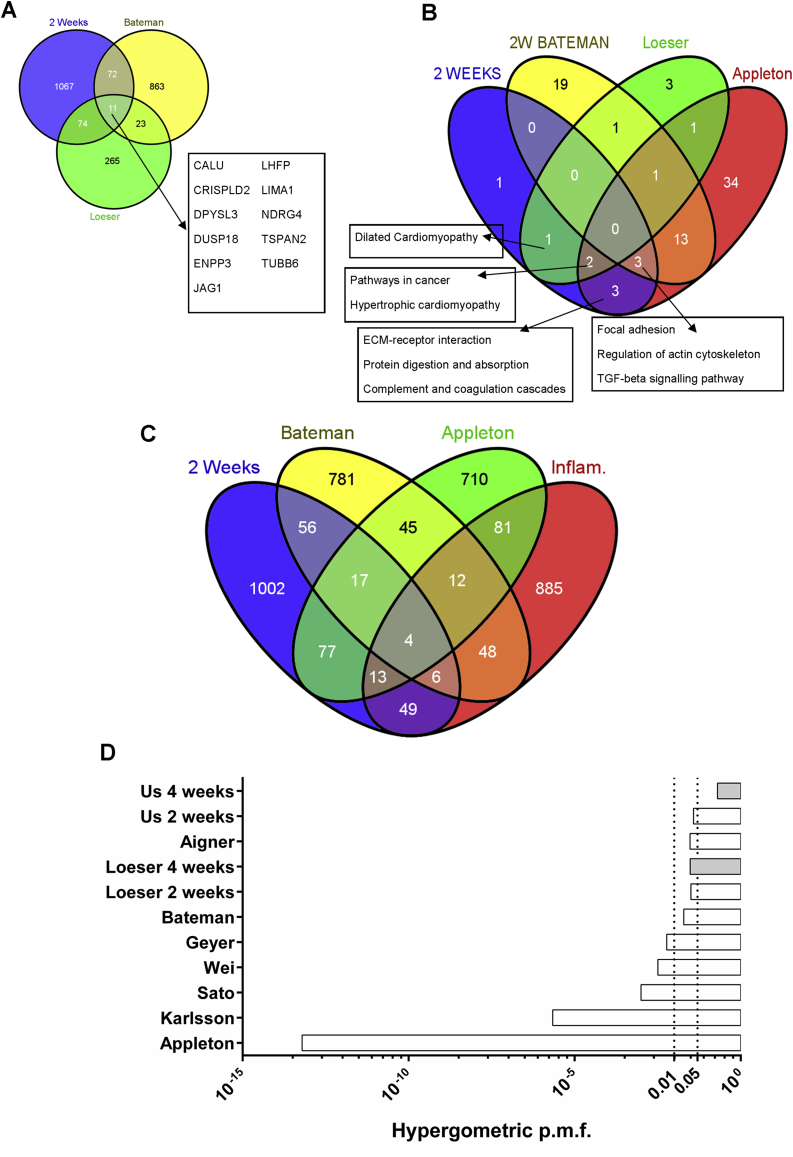
Gene and pathway overlap among our data and previously published microarrays in mice undergone DMM. A. Venn diagram showing the overlap between gene-sets of significantly regulated genes 2 weeks post-DMM (Blue: our results, Yellow: on RNA from microdissected tibial plateau cartilage over SHAM operated mice from Bateman *et al*., 2013^14^, Green: dataset from whole medial knee joint over SHAM operated mice from Loeser *et al*., 2013^17^). The inset contains the list of 11 genes that are differentially regulates all three studies. B. Venn diagram depicting the overlap for KEGG pathways that were significantly enriched in four different studies in rodents. (Blue: Current study, 2 weeks; Yellow: Bateman *et al*., 2013^14^ as in A; Green: Loeser *et al*., 2013^17^, as in A; Red: genes regulated in the rat femoral and tibial cartilage at 4 weeks post-anterior cruciate ligament transection and partial medial meniscectomy over SHAM, Appleton *et al*., 2007^13^.) Insets contain the list of the overlapping KEGG pathways for each comparison. C. Venn diagram showing the association between gene-sets of significantly regulated genes following surgically-induced OA in rodents and inflammation related genes. (Blue: Current study, 2 weeks; Yellow: Bateman *et al*., 2013^14^ as in A; Green: Appleton *et al*., 2007^13^, as in B; Red: a set of inflammation-related genes identified bioinformatically, Loza *et al*., 2007^18^.) D. Hypergeometric distribution p.m.f. for the associations of the nine arrays study here with the inflammatory gene-set from Loza *et al*., 2007, as in C. Dotted lines depict probabilities of 0.01 and 0.05.

As our study did not include any sham-operated controls, we compared our 2-week dataset with previously reported datasets from studies on mice 2 weeks post-DMM [Fig. 5(A)]. We observed an overlap of 86 genes with the 2-week Bateman dataset (*P* < 0.0001) and the same number of genes with the Loeser dataset (*P* < 0.0001). There were 33 genes in common between the Bateman and Loeser studies (*P* < 0.0001). We also identified 11 genes in common between the three datasets [Fig. 5(A) insert]. These include *Jag1*, a ligand for *Notch* receptor. By comparing the KEGG pathways enriched in each dataset [Fig. 5(B)], we show common pathways (focal adhesion, regulation of actin cytoskeleton and TGF-β signalling) in three out of the four microarrays, all studies using pure cartilage. We also compared our dataset and eight other arrays with a list constructed by genes involved in the inflammation process^18^ (Fig. 5). We observed 72 genes at 2 weeks (*P* = 0.03) involved with inflammation [Fig. 5(C) and (D)] and 4 genes at 4 weeks (*P* = 0.2) [Fig. 5(B)].

**Fig. 6:**
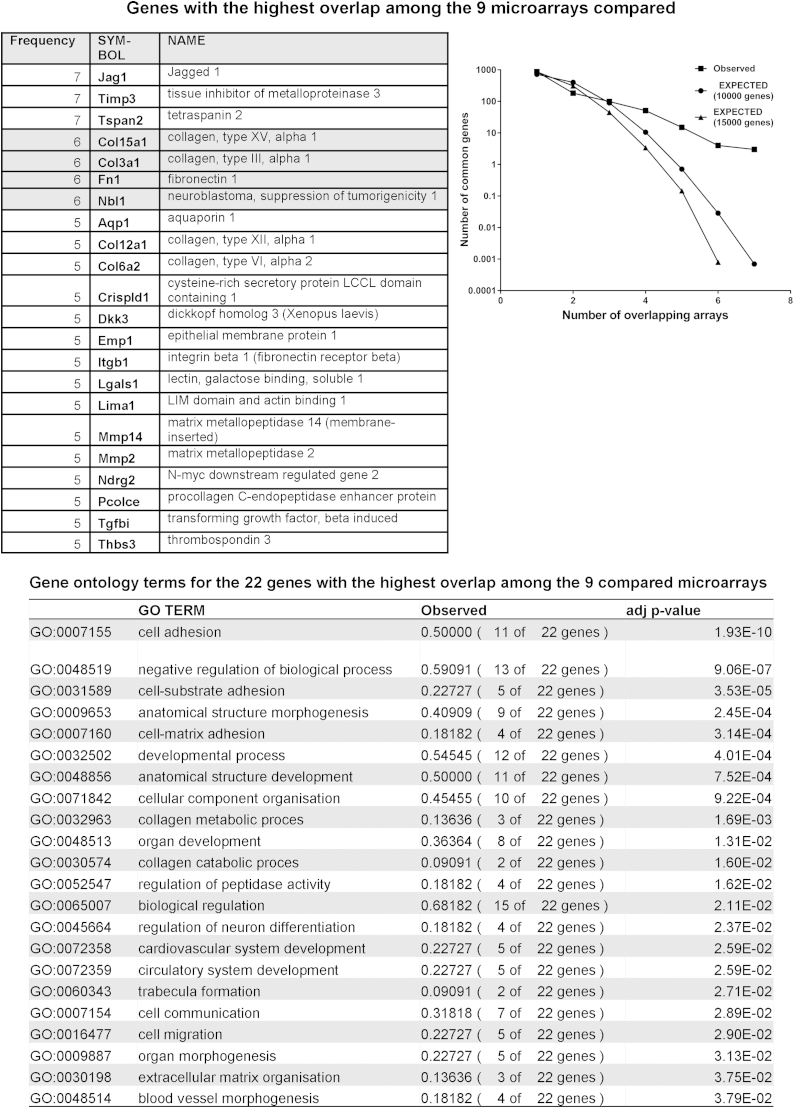
Comparison of amount of overlap between our study and previously published microarrays on cartilage from surgically induced OA in rodents and in OA human samples. Frequency histogram (squares) of the genes regulated in the current study and in eight previously published microarrays^6,7,13–17,43^. The expected number of genes was calculated by running a customised simulation of overlap between random samplings equal to the size of the compared array sets, without substitution and starting either from a sampling group of 10,000 (circles), or 15,000 (triangles) genes in MATLAB. The top table contains the genes with the highest frequency of occurrence among the gene sets studied. The bottom table contains the information on Biological Process gene ontology terms that were significantly overrepresented among the genes with the highest frequency of occurrence.

We compared our array with eight other arrays (Fig. 6) in order to identify genes deregulated in OA regardless of species, time and method of induction. At 2 weeks, 344 (28% of the regulated genes) were represented in at least one other microarray. There were no genes common to all arrays. The predicted overlap between our array and five or more arrays is 0.74 genes, from an initial pool of 10,000 elements after 10,000 simulations. However, we observed 22 genes overlapping in five or more arrays. GO slim analysis for the biological function of these 22 genes reveals that 50% of them are involved with cell adhesion and 54.5% of them in developmental processes (Fig. 6).

## Discussion

This study provides new insights into the localisation and temporal progression of gene expression. We used a novel micro-dissection technique allowing us to examine the MTP cartilage in isolation at a variety of time points post-disease induction. For the first time we demonstrate differential expression patterns in the medial vs the lateral tibial plateau. We identified a new molecule persistently up-regulated at every time point (*Tnn*, tenascin N) and modelled the evolution of gene changes as a network of potential protein–protein interactions, identifying *Fn1* as a central node at 2 weeks post-DMM. Finally, we presented an analysis showing commonality of disease-related genes across nine related studies irrespective of time, species and nature of OA induction. The study also provides important confirmatory evidence for a marked reduction in differential gene expression at 8 weeks, which was a surprising observation when recently reported by others^17^.

We studied the MTP cartilage as it degrades first^17,20^ mirroring the focal nature of cartilage lesions in human OA. Recent transcriptomic studies have used rat^13,15^ and mouse models^11,14,17,21,22^ to assess gene regulation in OA. Several studies focused on chondrocyte gene expression either by manual micro-dissection of cartilage^13,15,21^, or by laser capture^14^. These studies, along with our previous work^11^ have identified significant alterations in cartilage homeostasis in early disease. The significant overlap between pure cartilage and whole knee studies [Fig. 5(A)] highlights that cartilage contributes a significant proportion of the transcriptional changes although it constitutes a very small percentage of the joint. This study confirms the anatomical localisation of gene expression to areas of cartilage degeneration and strengthens the case for interrogating individual tissues of the joint.

A new finding was the persistent regulation of *Tnn* and *Fmod* at every time point. TNN is found in the kidney and sites of bone and smooth muscle development, including periosteum^23^. During development, it contributes to mineralisation of the mouse cavarial bones^24^. In contrast to *Tnc*, *Tnn* is down-regulated during differentiation of MC3T3-E1 mesenchymal cell (MSC) line to bone^25^. Both tenascins are regulated by BMP2 and the Wnt pathway^25^. Both tenascins and *Fmod* were strongly associated with chondrocyte dedifferentiation^26^. The up-regulation of *Fmod* and *Tnn* could be part of cartilage dedifferentiation in response to trauma, possibly as an adaptation or repair attempt. This could also explain the transcriptional changes we observed involving ECM remodelling, changes in actin cytoskeleton, attenuation of the muscle phenotype, deregulation of the TGF-β pathway and up-regulation of fibromodulin and tenascins C and N. The 22 genes identified in our microarray comparison further support this hypothesis as most are involved in development and cell adhesion (Fig. 6).

Fibromodulin is thought to regulate collagen fibril cross-linking, perhaps driving it towards a denser network^27,28^. The rapid accumulation of fibromodulin has been described in cartilage of late stage canine OA^28^. Both mRNA and protein for fibromodulin, biglycan and decorin are increased in human knee OA^29^. Fibromodulin, along with decorin and biglycan, bind to active TGF-β and release it when degraded. TGF-β1 also up-regulates fibromodulin expression, perhaps as a negative feedback loop to increase sequestration^30^. Interestingly, recombinant fibromodulin alone can reprogram human fibroblasts into multipotent cells^31^.

The TGF-β pathway was enriched in this study. At 2 weeks, *Tgfb2*, *Tgfb3*, *Ltpb1* (latent TGF-β binding protein 1) and *Ltpb4* were up-regulated but the receptors *Tgfbr1* and *Tgfbr3* were down-regulated. *Ltbp1* is involved in sequestration and activation of TGF-β^32^. Gremlin 1 (*Grem1*), an antagonist of TGF-β and the Wnt signalling pathway, was strongly down-regulated at 2 weeks. Gremlin 1 and FRP were identified as ‘natural breaks’ on hypertrophic differentiation of articular chondrocytes^33^. TGF-β is known to play a key role in osteophyte formation^34^ and two recent papers present conflicting roles of TGF-β in cartilage degradation^35,36^. Transgenic expression of TGF-β1 in murine osteoblasts induces OA and inhibition of TGF-β1 by antibodies or by deletion of TGF-β type II receptor, in nestin-positive MSCs, leads to less pronounced cartilage degradation after surgery-induced OA^35^. Conversely, deletion of TGF-β type II receptor in articular chondrocytes, induced progressive OA^36^. TGF-β action within a knee joint appears to be tissue dependent. TGF-β activity in the subchondral bone or in bone marrow MSCs might promote OA through bone remodelling but in the cartilage TGF-β is required for the maintenance of the chondrocyte phenotype. Levels outside the normal physiological range may promote cartilage degeneration.

Contractile elements formed another enriched group in this study. They were down regulated at both 2 and 4 weeks. Previous studies showed altered expression of contractile genes^21,22^. In STR/Ort mice knee cartilage they were higher in aged STR/Ort mice compared to control mice of the same age^21^ and these genes were down-regulated as the cartilage aged. A previous study identified *Myod1*, *Actc1*, *Actn2*, *Tnnc* and *Tnnt1* to be down-regulated over the time of differentiation to a hypertrophic phenotype^37^. Similarly to our study, genes associated with a muscle phenotype were down-regulated in the DMM-operated knees in the rat^13^. When comparing post-traumatic OA in old and young mice^22^, the contractile genes were down in the young cohort and up in the old one. They were also lower in the old sham-operated knees compared to the young sham-operated. The regulation of those genes appears to be dependent on the age of the tissue at injury.

*Dio2*, a gene linked with susceptibility to OA in humans was induced at 2 weeks^38^. Dio2 protein was expressed at higher levels in OA cartilage^39^. Interestingly, *Pthr1*, a receptor for the OA susceptibility gene *Pthlh* was also induced at 2 weeks^40^. Increased Dio2 expression, by altered methylation of the risk allele, leads to lower ECM deposition and induction of catabolic and mineralisation markers^41^, providing an explanation for epigenetic effects on the risk allele, leading to induction of the *Dio2* gene and an OA-like phenotype^41^.

A marked decrease in gene expression at 8 weeks following DMM surgery, with a subsequent increase at 16 weeks, was observed in the whole knee^17^. Our study supports this observation at 8 weeks. One might argue that the chondrocytes reach a new state that is almost indistinguishable at the gene level from the chondrocytes on the unoperated limb, apart from the persistence of *Fmod* and *Tnn*. This might reflect a transient adaptation to the altered biomechanical environment, which cannot be maintained in the presence of continuous biomechanical changes. Therefore possibly reflecting a point of no return if the abnormal biomechanical status persists. If true, it might be the last point of therapeutic intervention and requires further investigation.

We reconstructed interactions between regulated genes at 2 and 4 weeks. This revealed pathogenic elements including the potential interplay between ECM components and growth factors, the complement and coagulation systems, changes in focal adhesion and integrins and changes to the cytoskeleton. At 2 weeks, fibronectin (*Fn1*) was identified at the centre of the main subnetwork, consistent with previous studies^5,6,14,21^.

Olex *et al*.^42^ recently identified signalling and metabolic pathways post-DMM in mice by reconstructing networks based on their previous microarray data^17^. Among the affected pathways are ECM-receptor interaction, focal adhesion and the TGFβ signalling pathway, in agreement with our results. They also show involvement of the Wnt and hedgehog signalling pathways and riboflavin metabolism. Although we did not map the latter pathways, many of their constituent genes are dysregulated in our study. These include *Sfrp2* and *4*, *Fzd6* and *10*, *Wif1*, *Wisp1 Wisp2*, *Gli1*, *Ptch1* and *Enpp2* and *3*.

We compared our results with a recent microarray study of the tibial articular cartilage after DMM^14^. Eighty-six regulated genes overlapped at the 2-week time point [Fig. 5(A)]. Enriched pathways included the TGFβ signalling pathway (*Dcn*, *Thbs3*, *Bmp6*). Fibronectin, the key hub in our 2-week network, was also present. Comparison of commonly regulated genes among other studies, using the DMM model in the mouse^17^ or rat^13^, revealed TGFβ, regulation of the actin cytoskeleton and focal adhesion as the three commonly dysregulated pathways [Fig. 5(B)]. Previously a panel of 20 commonly regulated genes between rat and human OA was identified^15^. The same panel of genes showed 16 of those genes dysregulated in the mouse cartilage at any one time point^14^. We show agreement for 12 of the 20 genes (*Aqp1*, *Col3a1*, *Col4a1*, *Col6a2*, *Lum*, *Mmp3*, *Nbl1*, *Ndrg2*, *Pcolce*, *Timp1*, *Timp3*, *Tubb2b*). This motivated us to identify the overlap between our study and other microarrays in human samples. We have identified 194 genes in common with^16^, 88 genes with^7^, 33 with^43^ and 19 with^6^ (*P* < 0.0001 for all those comparisons). Three genes were up-regulated in all the human microarrays and ours (*Col15a1*, *Fn1* and *Timp3*). KEGG pathways common to these arrays include ECM and focal adhesion; TGFβ signalling is common to two datasets [Figs. 5 and 6]. Some notable genes with high coverage between arrays, like *Ltbp2* and *Col5a1* (in 7/9 examined), *Inhba* (in 6/9), *Serpine1* (5/9) and *Adamts5* (4/9) were not detected in our array. However, we are able to detect either homologues of those genes (*Ltbp1* and *4*, *Col5a2* and other Serpins) or changes in their pathways (TGFβ for *Ltbp2* and *Inhba*). It is difficult to attribute the undetectability to either a methodological or biological bias. We expect that both sources of variation contribute equally. However, for *Adamts5* we believe this to be mainly due to biological variation, given the poor repeatability among the various studies (4/9) and that we can only detect it very early after DMM (6-h) only by qPCR. Generally, the different microarray platform chosen, in combination with different amplification and labelling methods is probably the key factors behind gene-undetectability. This is a well-known problem concerning meta-analysis of microarray studies and so far no satisfactory way to tackle this problem exists^44–46^. A way to address this problem is to compare pathways that are dysregulated, rather than actual genes.

A limitation of this study is the use of contralateral knees rather than sham-operated controls. Although this reduced our animal use by 50%, it created two main issues. First, changes in the joint might be related to surgery itself, increasing the likelihood of false positives. Second, there may be changes occurring in the contralateral joint as a result of ipsilateral disease, perhaps driven by possible systemic factors or altered loading owing to the operated ipsilateral joint. Importantly, there was no statistical difference in histological scores or gene expression in the contralateral joints over time. Moreover, there is significant overlap between genes regulated in our study and those of other studies that included a sham control. Our histological observations and our previous work investigating pain following DMM show neutrophil infiltration in the joint cavity and synovitis is present post-operatively in the joints of both sham and DMM animals; this is normally acute and dissipates after the first week^47^. Comparing our dataset to a group of inflammation related genes^18^, shows that although there is a significant overlap between our 2-week dataset and the inflammatory one, the p.m.f. of this overlap is a lot higher than those observed for the datasets that originated against a sham control or healthy human cartilage samples [Fig. 5(D)]. In other words, cartilage produces inflammatory-related genes that are involved in the disease process. The number of those inflammatory genes appears to diminish at 4 weeks in the cartilage, as the p.m.f. increased to 0.2 indicating that such an overlap could be due to chance [Fig. 5(D)].

The examined transcriptome data indicate changes to the transcriptional wiring that affect:

1) ECM organisation
2) cell adhesion
3) TGFβ (and other signalling pathways like Notch, FGF and IGF), and
4) chondrocyte differentiation.

However, we are still unaware how these events are related to each other and how the altered mechanical status and the ageing process affect these components.

This study reinforces the transcriptomic approach to understanding disease pathogenesis and the relative importance of chondrocyte-driven changes in gene expression.

## Author contributions

• Conception and design: MDG, TLV, GBG, JS, HN & AC
• Analysis and interpretation of the data: MDG & AC
• Drafting of the article: MDG, TLV, AC
• Critical revision of the article for important intellectual content: All authors
• Final approval of the article: All authors
• Statistical expertise: AC
• Obtaining of funding: TLV, JS, HN
• Administrative, technical, or logistic support: GBG
• Collection and assembly of data: MDG, CD, AB, AC

## Role of the funding sources

The work was funded by NIAMS/NIH grant AR40994, by the medical engineering solutions in OA initiative (Wellcome Trust and the Engineering and Physical Sciences Research Council, Grant number 088844/Z/09/Z) and Arthritis Research UK, Centre for Osteoarthritis Pathogenesis, Grant number 20205. MDG was a PhD student, funded by scholarships from The Kennedy Trust for Rheumatology Research and the Royal College of Surgeons of England. The funding bodies had no role in the study design, collection, analysis and interpretation of data, nor in the writing of the manuscript, or in the decision to submit the manuscript for publication.

## Competing interests

None of the authors has any competing interests to declare.
