# Supplementary material for: Transcriptional analysis of micro-dissected articular cartilage in post-traumatic murine osteoarthritis
Source: Osteoarthritis Cartilage. 2015 Apr;23(4):616–28. doi: 10.1016/j.joca.2014.12.014 (PMC4373757; doi:10.1016/j.joca.2014.12.014)
Supplement: Supplementary file 1 [file mmc1.docx]

**Supplementary Materials and Methods**

*Microdissection protocol, cartilage RNA extraction and whole genome expression analysis (WGA)*

The operated and contralateral (control) joints were dissected from the hind limb. The musculature was removed and the collateral and cruciate ligaments divided to separate the joint. The menisci were removed and cartilage was dissected under a microscope. Cartilage from the medial and lateral tibial plateaus of both knees was collected separately in RNAlater (Invitrogen), frozen on dry ice and stored at -80 °C. The epiphysis was separated from the metaphysis by inserting the scalpel into the physis and twisting. For whole joint RNA extraction, the skin and musculature was removed and the femoral bone was cut at the proximal patella and the tibia was cut just above the insertion of the tibio-patellar ligament. Bone, meniscus and whole joint specimens were immediately snap frozen in liquid nitrogen.

Eight individual tibial plateau cartilage samples, frozen in RNAlater, were pooled together. The RNAlater was thawed and removed by pipette. The cartilage was disrupted using a #15 scalpel in Buffer RLT containing 1% beta-mecaptoethanol and homogenised using a PowerGen Model 125 homogeniser (Fisher Scientific, Loughborough, UK) on ice, for three 10 s intervals interrupted by 5 s breaks. The homogenised tissue was digested for 30 min at 55 °C with proteinase K. The supernatant was collected following centrifugation and added to 0.5 volumes of 100% ethanol. This mixture was added to an RNEasy MinElute spin column and the manufacturer’s instructions followed. Total RNA was eluted in 12 µL of RNase-free water. The RNA quality was determined using an Agilent 2100 Bioanalyzer with RNA 6000 Pico or Nano LabChip kits (Agilent Technologies) following the manufacturer’s instructions.

WGA was performed using the NuGEN Ovation Pico System (NuGEN). The cDNA was labelled using the FL-Ovation cDNA Biotin Module V2 (NuGEN). The quality and quantity of the amplified cDNA was checked using an Agilent 2100 Bioanalyzer with RNA 6000 Nano LabChip kit. The labelled cDNA was hybridised to an Illumina Mouse WG-6 v2.0 Expression BeadChip (Illumina). The allocation of samples to each BeadChip was randomised to minimise batch effects. GenomeStudio software (Illumina) was used to produce data for subsequent WGA. Data were loaded from GenomeStudio into R environment (*lumi*) [[1](#_ENREF_1)] for statistical analysis. Following quantile normalisation, *lumi* software was used to perform the probe selection and quantile normalisation on a log scale across all arrays [[2](#_ENREF_2)]. Independent pairwise comparisons and two-way ANOVA were completed using *limma* [[3](#_ENREF_3)].

DAVID [[4](#_ENREF_4)] was used to perform pathway analysis (KEGG) and functional annotation clustering (FAC) of the dysregulated genes sets. The Illumina BeadChip file (Mouse WG-6_V2_0_R2_11278593_A) was used as background list. A high stringency was used for the clustering with an EASE score greater than 0.1.

*Construction of regulatory pathways*

Genes found to be significantly regulated in the pairwise comparisons at 2, 4 and 8 weeks were analysed by EGAN 1.4 (Exploratory Gene Association Network) to reveal a protein-protein interaction network [[5](#_ENREF_5)]. Only the protein-protein interaction data in the EGAN database were used to feed the network. The constructed networks were verified in STRING version 9.05 [[6](#_ENREF_6)]. The prior knowledge utilised from STRING to reconstruct the networks included all databases apart from the ones that described gene neighbourhoods and gene fusion. Only interactions with a score greater than 0.9 were utilised and only the relationships that were present in STRING were kept to produce a network using cytoscape [[7](#_ENREF_7)].

*Comparison of previously published microarray gene sets*

The collected gene sets were translated to the corresponding mouse entrez gene ids to correct for differences in nomenclature between genes using the MGI batch query [[8](#_ENREF_8)] tool. Comparisons were made with customised tools in Excel and Matlab R2013b (Mathworks Inc, Cambridge UK). As we are unsure of the number of genes that are conserved between the human and mouse genomes, we decided to run simulations based on an initial pool of 10000 or 15000 elements. Each element corresponds to a gene. We created arrays of equal size as the eight arrays we chose to compare, drawn from an initial pool of 10000 or 15000 elements, randomly and without substitution. The arrays generated were compared to an array of equal size to the amount of significant genes at two weeks in our study (1224 elements). The amount of common elements between this array and any of the other eight arrays was determined. We performed 10000 iterations of this process in order to establish whether the observed overlap of the eight arrays with our study could be due to chance. The MATLAB code is available on request.

We decided to compare the 2-week time point with other microarray datasets because:

1. We were interested in identifying changes that are not due to the surgery alone. The major limitation of our study is the lack of a sham control. We hypothesised that genes overlapping between different datasets would help us uncover those changes that are not due to the operation but due to disease progression.
2. It was the most abundant of our datasets.
3. Of the available microarray data in the literature. Bateman’s study, which should theoretically more closely resemble ours, as it is from laser microdissected cartilage, has no 4 or 8 weeks. The only study to cover all the time points and it will be very useful as a comparison is Loeser’s. We have compared the 76 genes that we see change in our dataset at 4 weeks to those of Loeser’s and Appleton’s. We identify 11 genes overlapping with Appleton and 6 with Loeser. In addition, 32 of the genes at 4 weeks were represented in at least another dataset, when we added the human arrays and Wei’s. From the 6 overlaping with Loeser, FMOD and CRISPLD2 had at least one more representation in a human array, and from the 11 genes common with Appleton, MMP2, LUM, CTHRC1, CD74, WISP2, FAP and COL5A2 had at least a human counterpart.

As our microarray study lacked sham operated controls we were required to identify whether the dysregulated genes in our study could have arisen as a result of inflammatory processes. Given the lack of published microarray studies on cartilage or whole knee joint from models of inflammatory arthritis we decided to use as a test dataset a gene-set that included key genes that are involved on all aspects of inflammation and associated pathways [[9](#_ENREF_9)]. The human counterpart counts 1027 genes in the set that after correcting for the mouse homologues we ended up with 1098 genes. In order to calculate the hypergeometric distribution probability mass function for the observed overlap against this gene-set, we assumed a background of 20000 genes.

*Statistics*

All groups of data were checked for approximation to the Gaussian distribution using the D’ Agostino and Pearson omnibus normality test. Distributions were considered to be Gaussian if the p value for the null hypothesis was greater than 0.05. The summed histological scores for the contralateral tibial plateau cartilage (Fig. 1D) did not fit a Gaussian distribution. Therefore we transformed the values by taking the square root. This suppressed the skewness and kurtosis and the transformed values approximated a Gaussian distribution. A 2-way ANOVA was performed on the transformed values.

Where multiple comparisons between multiple endpoints or multiple genes were made, family-wise multiplicity corrections were applied with α = 0.05. Hence, all the reported p values are multiplicity adjusted p values.

For the microarray the moderated t-statistic derived p-values, were corrected for multiple testing by using the Benjamini-Hochberg method to control the false discovery ratio below the set threshold of 5%.

The tests performed at each case are described in the text and in the figure legends. The number of experimental units is given as n and the number of animals also provided when pooling of samples occurred. For example, for the microarrays we have 3 experimental units for each group at each time point, even though each experimental unit is the result of pooling the medial tibial plateau of eight animals.

To derive the number of animals required for each experiment, we performed power calculations based on previous data. For the histological data, we determined that 9 animals would be sufficient to enable us to discriminate 80% of the differences greater than 13 between treated and untreated animals. Indeed, the calculated power for our experiment to distinguish a difference between means greater than 2.5 was at least 85% for a value of α = 0.05 for the whole joint histology scores. For the individual compartment scores we have a power greater than 85% for a value of α = 0.05 to see differences of at least 3.5 between the means. For the microarrays, we based our power calculations on previous PCR data. We expected a standard deviation of 0.4, expecting about 2500 differentially expressed genes with an estimated number of false positive genes set to 5%. We wanted to be able to see at least 80% of the genes that changed at least 2 fold. This gave us an estimated number of 3 experimental units per experimental group with a per gene value of α = 0.05. The calculated power for our microarray study was greater than 90% for detecting changes greater than 2 with an FDR of 5%. All microarray power calculations were done according to [[10](#_ENREF_10)].

Statistical tests were performed on Graphad Prism, version 6, unless otherwise stated.


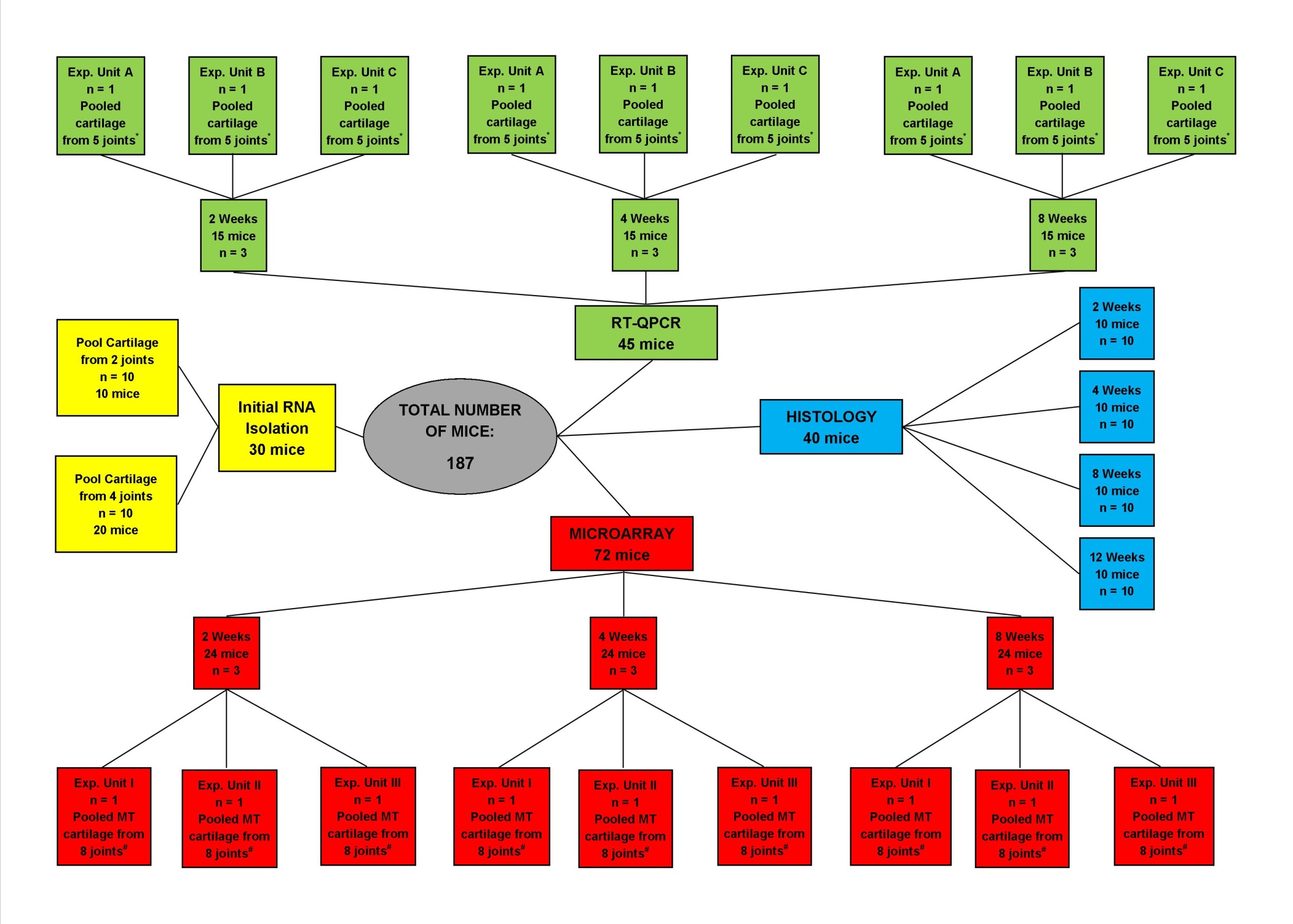


**Supplementary Figure 1**: A schematic representation of the animals used in this study. *: For the PCR experiment, the cartilage from the contralateral medial and lateral tibial plateau and the ipsilateral medial and lateral tibial plateau were dissected separately. The cartilage from the same anatomical compartment of 5 mice was pooled together. This translates as 3 time points, 2 experimental (CL vs IL) conditions and 2 anatomical positions (medial vs lateral tibial plateaus), 12 experimental groups all together with n = 3 for each group.

#: For the microarray study, the cartilage from the contralateral and the ipsilateral medial tibial plateau was dissected separately and the cartilage of 8 mice was pooled together. This translates as 3 time points, 2 experimental conditions (CL vs IL) and one anatomical position (medial tibial plateau), 6 experimental groups all together with n = 3 for each group.

**Table S1**. Summary of BeadChip data for statistical analysis. The estimated numbers of false positives are based on a false discovery rate (FDR) of 5%. Multiple probes may represent a single gene.

| **Comparisons** | **No. of probes selected** | **No. of probes differentially expressed (*p* < 0.05)** | **Estimated no. of false positives** |
| --- | --- | --- | --- |
| **2 wk control vs DMM** | 19600 | 1425 | 71 |
| **4 wk control vs DMM** | 19283 | 90 | 4 |
| **8 wk control vs DMM** | 20524 | 3 | - |
| **4 wk DMM vs 2 wk DMM** | 19763 | 522 | 26 |
| **8 wk DMM vs 2 wk DMM** | 20595 | 203 | 10 |

**Table S2**. Number of probes up, or down-regulated in DMM cartilage at 2, 4 and 8 weeks. Pairwise comparisons (*t*-test; adjusted p-value < 0.05). Some genes are counted more than once owing to the presence of multiple probes.

|  | **2 weeks** |  | **4 weeks** |  | **8 weeks** |  |
| --- | --- | --- | --- | --- | --- | --- |
| **Fold change** | **Up** | **Down** | **Up** | **Down** | **Up** | **Down** |
| **All significant** | 698 | 727 | 41 | 49 | 3 | 0 |
| **> 1.5** | 653 | 683 | 41 | 49 | 0 | 0 |
| **> 2** | 248 | 154 | 32 | 43 | 3 | 0 |
| **> 3** | 41 | 31 | 8 | 21 | 0 | 0 |
| **> 4** | 11 | 22 | 3 | 13 | 0 | 0 |
| **> 5** | 3 | 11 | 1 | 4 | 0 | 0 |
| **> 6** | 2 | 6 | 0 | 1 | 0 | 0 |
| **> 7** | 0 | 1 | 0 | 0 | 0 | 0 |

**Table S3**.  Most significantly regulated genes in DMM cartilage at 8 weeks.

| **ProbeID** | **ACCESSION** | **SYMBOL** | **logFC** | **adj.P.Val** |
| --- | --- | --- | --- | --- |
| 5080358 | NM_021355.2 | Fmod | 1.94629 | 0.025639 |
| 4180626 | NM_177839.2 | Tnn | 1.67216 | 0.027241 |

**Table S4**. KEGG pathways associated with differentially regulated genes at 2 and 4 weeks post DMM.

| **2 WEEKS** | | | | |
| --- | --- | --- | --- | --- |
| **Term** | **Count** | **%** | **P Value** | **Genes** |
| ECM-receptor interaction | 26 | 2.89 | 6.10E-14 | TNC, COL3A1,ITGA11, ITGA10, SDC4, ITGB1, SDC2, CHAD, GP9, CD44, COL6A2, COL6A1,TNN,THBS1,THBS2,THBS3,FN1,COL4A2,COL4A1,COL5A3,COL5A2,ITGA9,SDC1,LOC640441,LAB1-1,RELN,LAMC1 |
| Focal adhesion | 34 | 3.78 | 4.10E-10 | TNC, COL3A1, ITGA11, ITGA10, ITGB1, CHAD, DOCK1, BCL2, COL6A2, COL6A1, TNN, THBS1, THBS2, THBS3, FN1, COL4A2, GM3655, COL4A1, VAV3, RAF1, MYLK2, MYLPF, IGF1, ACTN1, ACTN3, COL5A3, FLNC, COL5A2, VEGFC, ITGA9, LOC640441, LAMB1-1, PDGFRA, MAPK8, RELN, LAMC1 |
| Complement and coagulation cascades | 15 | 1.67 | 1.57E-05 | C3AR1, C7, LOC100048401, LOC677441, GM5196, CBX3, C1S, LOC100048018, SERPINA1B, SERPINA1A, SERPINA1D, CFH, GM7721, CFD, KNG1, GM5792, C5AR1, LOC633016, SERPING1, GM8893, SERPINA1E, GM6917, C1QB, THBD, GM6901, LOC632383, GM7469, F2R |
| TGF-beta signaling pathway | 14 | 1.56 | 3.06E-04 | BMP4, PPP2R1B, LTBP1, ACVRL1, TGFBR1, TGFB3, DCN, TGFB2, LOC640441, ID4, THBS1, THBS2, BMP5, THBS3, BMP6 |
| Pathways in cancer | 31 | 3.45 | 7.06E-04 | FGFR3, EGLN3, TGFB3, MMP2, ITGB1, GLI1, TGFB2, ARNT, BCL2, RUNX1, TPR, FGF2, TRAF4, FN1, BMP4, LOC100043998, COL4A2, GM3655, COL4A1, EPAS1, MSH2, TGFBR1, RAF1, IGF1, FZD6, VEGFC, FZD10, RASSF1, LAMB1-1, PDGFRA, MAPK8, PTCH1, LOC100048537, LAMC1 |
| Regulation of actin cytoskeleton | 22 | 2.45 | 0.001932 | LOC100047211, FGFR3, VAV3, WASF1, ITGA11, RAF1, MYLK2, MYLPF, ITGA10, ACTN1, ACTN3, ITGB1, IQGAP1, ITGA9, DOCK1, GSN, SCIN, CYFIP2, PDGFRA, TMSB4X, FGF2, F2R, FN1 |
| Hypertrophic cardiomyopathy (HCM) | 12 | 1.33 | 0.002389 | ACTC1, LMNA, ITGA11, TGFB3, IGF1, ITGA10, TPM2, TTN, ITGB1, TPM4, TGFB2, ITGA9, LOC100048431, GM7809 |
| Dilated cardiomyopathy | 12 | 1.33 | 0.005024 | ACTC1, LMNA, ITGA11, TGFB3, IGF1, ITGA10, TPM2, TTN, ITGB1, TPM4, TGFB2, ITGA9, LOC100048431, GM7809 |
| O-Glycan biosynthesis | 6 | 0.67 | 0.010425 | GALNT2, GALNT10, WBSCR17, GALNTL2, LOC635918, OGT, GCNT1, LOC676881 |
| Colorectal cancer | 10 | 1.11 | 0.027388 | FZD10, GM3655, MSH2, BCL2, TGFBR1, TGFB3, PDGFRA, RAF1, MAPK8, FZD6, TGFB2 |
| Heparan sulfate biosynthesis | 5 | 0.56 | 0.040139 | HS3ST3A1, EXT1, EXTL1, HS3ST3B1, HS2ST1 |
| Galactose metabolism | 5 | 0.56 | 0.040139 | AKR1B8, B4GALT1, PFKP, UGP2, GLB1 |
| Small cell lung cancer | 9 | 1.0 | 0.058254 | COL4A2, GM3655, COL4A1, BCL2, LAMB1-1, LAMC1, APAF1, ITGB1, TRAF4, FN1 |
| Bladder cancer | 6 | 0.67 | 0.059513 | VEGFC, FGFR3, LOC640441, RASSF1, RAF1, THBS1, MMP2 |
| Renin-angiotensin system | 4 | 0.44 | 0.059917 | NLN, ANPEP, CTSA, ENPEP |
| Gap junction | 9 | 1.0 | 0.065258 | TUBB2A-PS2, TUBB2B, TUBB2A, TUBB5, PDGFRA, RAF1, GJA1, TUBB6, ITPR3, PRKG1 |
| Lysosome | 11 | 1.22 | 0.068864 | LAMP2, CTSK, LAPTM5, IGF2R, LGMN, CTSC, CTSA, CTSB, CD63, CTSH, GLB1 |
| Arrhythmogenic right ventricular cardiomyopathy (ARVC) | 8 | 0.89 | 0.075467 | ITGA9, ITGA11, LMNA, GJA1, ACTN1, ITGA10, ACTN3, ITGB1 |
| Fc gamma R-mediated phagocytosis | 9 | 1.0 | 0.089285 | VAV3, MARCKSL1, GSN, MARCKSL1-PS3, WASF1, SPHK1, SCIN, RAF1, MARCKS, PPAP2A |
| **4 WEEKS** | | | | |
| **Term** | **Count** | **%** | **P Value** | **Genes** |
| Focal adhesion | 8 | 12.12 | 2.77E-05 | TNC, MYLK2, MYLPF, COL6A1, TNN, ACTN3, COL5A2, THBS3 |
| ECM-receptor interaction | 5 | 7.57 | 5.69E-04 | TNC, COL6A1, TNN, COL5A2, THBS3 |
| Hypertrophic cardiomyopathy (HCM) | 3 | 4.54 | 0.058201 | ACTC1, PRKAA2, TTN |

**Table S5**. Top ten clusters after functional annotation clustering analysis of genes up-regulated in DMM cartilage at two weeks. Group enrichment score orders the clusters. This is the geometric mean (-log scale) of member's *p*-values in the corresponding annotation cluster; cluster 1 is the most enriched.

| **Cluster** | **Category*^a^*** | **ID** | **Term** | **Study pop. %*^b^*** | **p-value*^c^*** |
| --- | --- | --- | --- | --- | --- |
| 1 | INTERPRO | IPR006025 | Peptidase M, neutral zinc metallopeptidases, zinc-binding site | 4.42 | 3.22E-14 |
| 2 | INTERPRO | IPR003129 | Laminin G, thrombospondin-type, N-terminal | 1.89 | 2.75E-08 |
| 3 | INTERPRO | IPR006210 | EGF-like | 4.42 | 2.85E-07 |
| 4 | GOTERM_MF_FAT | GO:0005520 | Insulin-like growth factor binding | 1.89 | 4.72E-08 |
| 5 | INTERPRO | IPR001881 | EGF-like calcium-binding | 3.16 | 8.03E-08 |
| 6 | GOTERM_MF_FAT | GO:0070011 | Peptidase activity, acting on L-amino acid peptides | 8.42 | 5.46E-08 |
| 7 | GOTERM_BP_FAT | GO:0001568 | Blood vessel development | 4.84 | 3.52E-07 |
| 8 | GOTERM_MF_FAT | GO:0001871 | Pattern binding | 3.16 | 5.82E-06 |
| 9 | INTERPRO | IPR018358 | Disintegrin, conserved site | 1.89 | 3.24E-05 |
| 10 | INTERPRO | IPR001007 | von Willebrand factor, type C | 1.68 | 6.40E-05 |

*^a^* Gene ontology (GO) terms were for cellular component (CC), biological process (BP) or molecular function (MF); INTERPRO, InterPro database ;PIR, Protein Information Resource.

*^b^* Percentage of the total genes submitted that are denoted by the annotation term.

*^c^* Modified Fisher Exact p-value (EASE score) [[11](#_ENREF_11)]. Perfect enrichment p = 0, the smaller the p-value the more enriched the term.

**Table S6**. Top 10 clusters from functional annotation clustering analysis of genes down-regulated in DMM cartilage at two weeks. Group enrichment score orders the clusters. This is the geometric mean (-log scale) of member's *p*-values in the corresponding annotation cluster; cluster 1 is the most enriched.

| **Cluster** | **Category*^a^*** | **ID** | **Term** | **Study pop. %*^b^*** | **p-value*^c^*** |
| --- | --- | --- | --- | --- | --- |
| 1 | GOTERM_CC_FAT | GO:0030017 | Sarcomere | 2.98 | 2.27E-06 |
| 2 | GOTERM_BP_FAT | GO:0030029 | Actin filament-based process | 3.52 | 1.34E-04 |
| 3 | GOTERM_BP_FAT | GO:0008219 | Cell death | 5.96 | 5.28E-04 |
| 4 | GOTERM_BP_FAT | GO:0031032 | Actomyosin structure organization | 1.36 | 7.89E-05 |
| 5 | PIR_SUPERFAMILY | PIRSF001630 | Serpin | 1.63 | 0.005 |
| 6 | GOTERM_BP_FAT | GO:0009725 | Response to hormone stimulus | 2.98 | 8.57E-04 |
| 7 | GOTERM_MF_FAT | GO:0004866 | Endopeptidase inhibitor activity | 2.44 | 0.011 |
| 8 | INTERPRO | IPR001212 | Somatomedin B | 0.81 | 0.014 |
| 9 | GOTERM_BP_FAT | GO:0043067 | Regulation, programmed cell death | 5.15 | 0.019 |
| 10 | INTERPRO | IPR018248 | EF hand | 2.17 | 0.001 |

*^a^* Gene ontology (GO) terms were for cellular component (CC), biological process (BP) or molecular function (MF); INTERPRO, InterPro database ;PIR, Protein Information Resource.

*^b^* Percentage of the total genes submitted that are denoted by the annotation term.

*^c^* Modified Fisher Exact p-value (EASE score) [[11](#_ENREF_11)]. Perfect enrichment p = 0, the smaller the p-value the more enriched the term.

**Table S7**. Top 10 clusters from functional annotation clustering analysis of genes down-regulated four weeks following DMM surgery. Group enrichment score orders the clusters. This is the geometric mean (-log scale) of member's *p*-values in the corresponding annotation cluster; cluster 1 is the most enriched.

| **Cluster** | **Category*^a^*** | **ID** | **Term** | **Study pop. %*^b^*** | **p-value*^c^*** |
| --- | --- | --- | --- | --- | --- |
| 1 | GOTERM_CC_FAT | GO:0030017 | Sarcomere | 25 | 1.47E-12 |
| 2 | GOTERM_CC_FAT | GO:0015629 | Actin cytoskeleton | 30.6 | 1.88E-12 |
| 3 | GOTERM_BP_FAT | GO:0030036 | Actin cytoskeleton organization | 16.7 | 1.94E-05 |
| 4 | INTERPRO | IPR002048: | Calcium-binding EF-hand | 16.7 | 7.82E-06 |
| 5 | GOTERM_BP_FAT | GO:0030239 | Myofibril assembly | 11.1 | 6.51E-06 |
| 6 | GOTERM_BP_FAT | GO:0014706 | Striated muscle tissue development | 13.9 | 1.20E-04 |
| 7 | GOTERM_MF_FAT | GO:0008092 | Cytoskeletal protein binding | 16.7 | 0.002 |
| 8 | GOTERM_MF_FAT | GO:0005200 | Structural constituent of cytoskeleton | 11.1 | 3.80E-05 |
| 9 | GOTERM_CC_FAT | GO:0016529 | Sarcoplasmic reticulum | 8.3 | 0.002 |
| 10 | GOTERM_MF_FAT | GO:0003774 | Motor activity | 8.3 | 0.037 |

*^a^* Gene ontology (GO) terms were for cellular component (CC), biological process (BP) or molecular function (MF); INTERPRO, InterPro database ;PIR, Protein Information Resource.

*^b^* Percentage of the total genes submitted that are denoted by the annotation term.

*^c^* Modified Fisher Exact p-value (EASE score) [[11](#_ENREF_11)]. Perfect enrichment p = 0, the smaller the p-value the more enriched the term.

**References**

1. Du P, Kibbe WA, Lin SM. lumi: a pipeline for processing Illumina microarray. Bioinformatics 2008; 24: 1547-1548.

2. Lin SM, Du P, Huber W, Kibbe WA. Model-based variance-stabilizing transformation for Illumina microarray data. Nucleic Acids Res 2008; 36: e11.

3. Smyth GK, Michaud J, Scott HS. Use of within-array replicate spots for assessing differential expression in microarray experiments. Bioinformatics 2005; 21: 2067-2075.

4. Dennis G, Jr., Sherman BT, Hosack DA, Yang J, Gao W, Lane HC, et al. DAVID: Database for Annotation, Visualization, and Integrated Discovery. Genome Biol 2003; 4: P3.

5. Paquette J, Tokuyasu T. EGAN: exploratory gene association networks. Bioinformatics 2010; 26: 285-286.

6. Franceschini A, Szklarczyk D, Frankild S, Kuhn M, Simonovic M, Roth A, et al. STRING v9.1: protein-protein interaction networks, with increased coverage and integration. Nucleic Acids Res 2013; 41: D808-815.

7. Lopes CT, Franz M, Kazi F, Donaldson SL, Morris Q, Bader GD. Cytoscape Web: an interactive web-based network browser. Bioinformatics 2010; 26: 2347-2348.

8. Eppig JT, Blake JA, Bult CJ, Kadin JA, Richardson JE, Mouse Genome Database G. The Mouse Genome Database (MGD): comprehensive resource for genetics and genomics of the laboratory mouse. Nucleic Acids Res 2012; 40: D881-886.

9. Loza MJ, McCall CE, Li L, Isaacs WB, Xu J, Chang BL. Assembly of inflammation-related genes for pathway-focused genetic analysis. PLoS One 2007; 2: e1035.

10. Lee ML, Whitmore GA. Power and sample size for DNA microarray studies. Stat Med 2002; 21: 3543-3570.

11. Hosack DA, Dennis G, Jr., Sherman BT, Lane HC, Lempicki RA. Identifying biological themes within lists of genes with EASE. Genome Biol 2003; 4: R70.
